# Overview and Discussion of the Competition on Legal Information, Extraction/Entailment (COLIEE) 2023

**DOI:** 10.1007/s12626-023-00152-0

**Published:** 2024-01-12

**Authors:** Randy Goebel, Yoshinobu Kano, Mi-Young Kim, Juliano Rabelo, Ken Satoh, Masaharu Yoshioka

**Affiliations:** 1 Department of Computing Science and Alberta Machine Intelligence Institute, University of Alberta, Edmonton, AB Canada; 2 Faculty of Informatics, Shizuoka University, Hamamatsu, Shizuoka, Japan; 3 Department of Science, Augustana Faculty, University of Alberta, Edmonton AB, Canada; 4 Alberta Machine Intelligence Institute, University of Alberta, Edmonton, Alberta Canada; 5 National Institute of Informatics, Chiyoda-ku, Tokyo, Japan; 6 Faculty of Information Science and Technology, Hokkaido University, Sapporo-shi, Hokkaido Japan

**Keywords:** COLIEE2023, Legal information retrieval, Legal information entailment

## Abstract

We summarize the 10th Competition on Legal Information Extraction and Entailment. In this tenth edition, the competition included four tasks on case law and statute law. The case law component includes an information retrieval task (Task 1), and the confirmation of an entailment relation between an existing case and a selected unseen case (Task 2). The statute law component includes an information retrieval task (Task 3), and an entailment/question-answering task based on retrieved civil code statutes (Task 4). Participation was open to any group based on any approach. Ten different teams participated in the case law competition tasks, most of them in more than one task. We received results from 8 teams for Task 1 (22 runs) and seven teams for Task 2 (18 runs). On the statute law task, there were 9 different teams participating, most in more than one task. 6 teams submitted a total of 16 runs for Task 3, and 9 teams submitted a total of 26 runs for Task 4. We describe the variety of approaches, our official evaluation, and analysis of our data and submission results.

## Introduction

The objective of the Competition on Legal Information Extraction/Entailment (COLIEE) is to encourage the development of state of the art for information retrieval and entailment methods using legal texts. It is usually co-located with JURISIN, the Juris-Informatics workshop series, which was created to promote community discussion on both fundamental and practical issues on legal information processing, with the intention to embrace many disciplines: these include law, social sciences, information processing, logic and philosophy, and the existing conventional "AI and law” area. In alternate years, COLIEE is organized as a workshop of the International Conference on AI and Law (ICAIL), which was the case in 2017 and 2019, 2021, and again in 2023. Until 2017, COLIEE consisted of two tasks: information retrieval (IR) and entailment using Japanese Statute Law (civil law). From COLIEE 2018, we introduced a new and challenging case law IR and entailment tasks based on Canadian case law.

Task 1 is a legal case retrieval task, and it involves reading a query case and extracting supporting cases from the provided case law corpus, hypothesized to be relevant to the query case. Task 2 is the legal case entailment task, which involves the identification of relevant paragraphs or paragraphs from existing cases, which entail a given fragment of a new case. Tasks 3 and 4 are statute law tasks that use questions from the Japanese Bar exam to judge whether the given statement is true or not. Task 3 is an information retrieval task that identifies relevant articles for the legal entailment (Task 4). Finally, Task 4 is a legal entailment task that judges whether the given statement is true or not. In contrast to COLIEE 2022, COLIEE 2023 introduced 319 new query cases for Task 1 and 100 for Task 2. Furthermore, for the test data of Task 3 and Task 4 in COLIEE 2023, 100 fresh questions sourced from the 2022 bar exam were used.

The rest of our paper is organized as follows: Sects. 2, 3, 4, 5, describe each task, presenting their definitions, datasets, list of approaches submitted by the participants, and results attained. Section 6 presents some final remarks.

## Task 1—Case Law Retrieval

### Task Definition

This task consists of finding which cases, among a set of provided candidate cases, should be "noticed” with respect to a given query case.^1^ More formally, given a query case *q* and a set of candidate cases $C=\{c_1, c_2,..., c_n\}$, the task is to find the supporting cases $S=\{s_1, s_2,..., s_n \mid s_i \in C \wedge \text {noticed}(s_i, q)\}$ where noticed$(s_i, q)$ denotes a relationship which is true when $s_i \in S$ is a noticed case with respect to *q*.

The official metric employed was the micro-average of the F1 score.

1 $$\begin{aligned} \text {Precision}= & {} \frac{\text {number}\ \text {of}\ \text {retrieved}\ \text {relevant}\ \text {articles}}{\text {number}\ \text {of}\ \text {returned}\ \text {articles}}, \end{aligned}$$

2 $$\begin{aligned} \text {Recall}= & {} \frac{\text {Number}\ \text {of} \ \text {retrieved}\ \text {relevant}\ \text {articles}}{\text {number}\ \text {of}\ \text {relevant}\ \text {articles}}, \end{aligned}$$

3 $$\begin{aligned} F1= & {} \frac{\text {precision} \times \text {recall}}{\text {precision} + \text {recall}}. \end{aligned}$$

### Case Law Dataset

The dataset consists of a total of 5735 case law files. Also provided is a labeled training set of 4400 cases, of which 959 are query cases. On average, the training data includes approximately 4.67 noticed cases per query case, which are to be identified among the 4400 cases. To prevent competitors from merely using existing embedded conventional citations in historical cases to identify cited cases, citations are suppressed from all candidate cases and replaced by a “FRAGMENT_SUPPRESSED” tag indicating that a fragment containing a citation was removed from the case contents.

A test set consists of a total of 1335 cases, with 319 query cases and a total of 859 true noticed cases (an average of 2.69 noticed cases per query case). Initially, the golden labels for that test set are not provided to competitors.

### Approaches

We received 22 submissions from 8 different teams for Task 1. In this section, we present an overview of the approaches taken by the 7 teams which submitted papers describing their methods. Please refer to the corresponding papers for further details.

- **IITDLI** [4] developed an approach to task 1 that can be summarized in 6 steps: (1) Pre-processing: remove French words, extract years, and perform feature extraction using unigram/word features; (2) Term extraction: use Kullback–Leibler Divergence for Informativeness and Term Frequency, and Inverse Document Frequency for query reformulation; (3) Retrieval: use BM25 as a ranking model to retrieve top-n results from the corpus; (4) Filtering: apply a year filtering method to refine the results; (5) Experiment with additional filters, which ended up not being used in the final submission because they showed inferior results in their experiments; and finally, (6) Post-processing: Implement a threshold scheme for selecting the final set of candidate relevant cases, which improves precision and overall F1 score.
- **JNLP (3 runs)** [2] applies data augmentation techniques to produce additional training data, then employs a variety of large language models (LLMs) to capture the nuances of legal language. The data augmentation step generates synthetic cases that exhibit similar attributes to the original cases. Subsequently, an LLM is trained on the augmented dataset and used to retrieve relevant cases (the same overall approach is also used to determine entailment in Task 2).
- **NOWJ** [14] proposes a two-phase matching approach ("mono matching”, at paragraph/decision level) and "panorama matching” (case level). The pre-processing step removes French content, segments cases into paragraphs, extracts case years, removes redundant characters, and detects "important” passages. In the mono-matching phase, lexical and semantic matching models are combined. Lexical matching was done with BM25 [13] to calculate relevance scores between paragraphs, while semantic matching was carried out using a fine-tuned model. A lexical model was initially used to narrow down the search space and select potential candidates. In the panorama matching phase, a Longformer model was used to compare base and candidate cases based on their overall similarities.
- **THUIR (3 runs)** [5] designs structure-aware pre-trained language models to enhance legal case understanding, via an encoder-decoder architecture that explicitly models the relationships between different structures and learns the legal knowledge implied in the structures through pretraining on a large number of legal cases. The authors also propose heuristic pre- and post-processing approaches to reduce the influence of irrelevant items. In the pre-processing step, they eliminate irrelevant information, extract a summary, and also extract reference sentences. For post-processing, they filter the candidate set based on the trial date and implement a dynamic cut-off to identify relevant cases for each query. Finally, they use learning-to-rank methods to merge features with different dimensions. They employ NDCG (normalized discounted cumulative gain) as the ranking optimization objective and select the model that performs best on the validation set for testing.
- **UA (3 runs)** [11] uses a transformer-based model to generate paragraph embeddings, and then calculates the similarity between paragraphs of a query case and positive and negative cases. These calculated similarities are used to generate feature vectors (10-bin histograms of all pair-wise comparisons between 2 cases) which are used by a Gradient Boosting classifier to determine if those cases should be noticed or not. The UA team also applies pre- and post-processing heuristics to generate the final results.
- **UFAM (3 runs)** [9] explores the idea of filtering + ranking results, which was implemented by topic discovery using BERTopic, followed by a ranking algorithm. The topic discovery step assigns *k* topics to a case (*k* being a parameter which is varied in the experiments). The ranking step takes whatever candidate contains the dominant query topic in its *k* most relevant topic list. The ranking was implemented in 3 different ways, the best of which was the cosine similarity between the query and a candidate case.

The other participating teams did not send papers describing the details of their approaches.

### Results and Discussion

Table 1 shows the results of all submissions received for Task 1 for COLIEE 2023. A total of 22 submissions from 8 different teams were evaluated. Similar to what happened in recent COLIEE editions, the F1 scores are generally low, which reflects the fact that the task is now more challenging than its previous formulation.^2^

Most of the participating teams applied some form of traditional IR technique such as BM25, transformer-based methods such as BERT, or a combination of both. The best-performing team (THUIR) employed pre-trained language models to enhance legal case understanding, pre- and post-processing heuristic approaches to reduce the influence of irrelevant items, and learning-to-rank methods at the end, to merge features with different dimensions.

**Table 1:** Task 1 results

Team	F1	Precision	Recall
THUIR	0.3001	0.2379	0.4063
THUIR	0.2907	0.2173	0.4389
IITDLI	0.2874	0.2447	0.3481
THUIR	0.2771	0.2186	0.3783
NOWJ	0.2757	0.2263	0.3527
NOWJ	0.2756	0.2272	0.3504
IITDLI	0.2738	0.2107	0.3912
IITDLI	0.2681	0.2063	0.3830
JNLP	0.2604	0.2044	0.3586
NOWJ	0.2573	0.2032	0.3504
UA	0.2555	0.2847	0.2317
UFAM	0.2545	0.2975	0.2224
JNLP	0.2511	0.1971	0.3458
JNLP	0.2493	0.1931	0.3516
UA	0.2390	0.3045	0.1967
UA	0.2345	0.2400	0.2293
UFAM	0.2345	0.3199	0.1851
UFAM	0.2156	0.3182	0.1630
YR	0.1377	0.1060	0.1967
YR	0.1051	0.0809	0.1502
LLNTU	0.0000	0.0000	0.0000
LLNTU	0.0000	0.0000	0.0000

Specific error analysis for Task 1 would require manual analysis of the whole dataset, which is not feasible. We have run an experiment to try and identify shallow features in the dataset that would correlate with the results. We have calculated the average F1 score per question, considering all teams’ submissions. The idea was to find out what are the more challenging cases overall, and what characteristics those cases share, if any. We calculated how the average F1 score for each query case in the test set correlates with two text features: (1) the query case length in bytes, and (2) the number of expected noticed cases in the golden dataset. The correlation values calculated were $-$0.0395 and 0.2189, respectively.

There is a very low correlation for (1), but the correlation for (2), although not high, is not irrelevant. We hypothesize that the F1-score tends to increase for cases which have more expected noticed cases because there is a higher chance for a system to get some prediction right. In a scenario where the scores are overall very low, having a better chance to get something right increases the average F1 score. To test this, we ran a second correlation analysis: we sorted the test dataset in descending order according to the average F1 score per query case and split the test dataset into four roughly equal parts (80, 80, 80, and 79 cases), and then calculate the correlation (2) for each one of those groups. The results, starting with the highest F1 scores to the lowest, were: 0.1286, $-$0.0748, $-$0.0569, 0.3994. The fourth group, i.e., the one with the lowest F1-scores, presents the highest correlation between the F1-score and the number of expected noticed cases, which seems to confirm our hypothesis that increasing the size of the expected noticed cases set has a more noticeable impact for those cases with a very low overall F1 score.

When it comes to the approaches used in this task, we can see some emerging trends, such as the combination of traditional IR methods with Large Language Models (LLMs). We also noticed the current edition presented an additional challenge, which was the shift in the noticed cases average from the training to the test datasets. Keeping those values close is a challenge because we rely on data provided by an external partner, which we do not fully control. Still, we intend to improve the sampling methods in order to keep the distributions in the training and test datasets as similar as possible. In the current edition, we were able to remove cases that had exactly the same contents but were represented as different files in the dataset. We intend to improve the method used to identify such cases to capture minor/immaterial changes in different file contents that are likely to represent the same case.

## Task 2—Case Law Entailment

### Task Definition

Given a base case and a specific text fragment from it, together with a second case relevant to the base case, this task consists of determining which paragraphs of the second case entail that fragment of the base case. More formally, given a base case *b* and its entailed fragment *f*, and another case *r* represented by its paragraphs $P=\{p_1, p_2,..., p_n\}$ such that noticed(*b*, *r*) as defined in section 2 is true. The task consists in finding the set $E=\{p_1, p_2,..., p_m \mid p_i \in P\}$ where entails$(p_i, f)$ denotes a relationship which is true when $p_i \in P$ entails the fragment *f*.

### Dataset

In Task 2, 625 query cases and 22,018 paragraphs were provided for training. There were 100 query cases and 3,765 paragraphs in the testing dataset. On average, there are 35.22 candidate paragraphs for each query case in the training dataset, and 37.65 candidate paragraphs for each query case in the testing dataset. The average number of relevant paragraphs for Task 2 was 1.17 paragraphs for training and 1.2 paragraphs for testing. The average query length is 35.36 words in the training set and 36.57 in the test set. The average candidate length is 102.32 words in the training set and 104.71 in the test set.

### Approaches

Seven teams submitted a total of 18 runs to this task. Here, we introduce six teams’ approaches that described their methods in more detail in their respective papers. One team has not submitted their paper to the COLIEE 2023 workshop.

- **CAPTAIN (3 runs)** [7] proposes an approach based on the pre-trained monoT5^3^ sequence-to-sequence model, which is fine-tuned with hard negation mining and ensembling techniques. MonoT5 is a novel approach to document ranking by fine-tuning the pre-trained T5 models with modified training data for the point-wise classification task, which outperformed the traditional BERT re-ranker approach. The approach uses a straightforward input template to represent the point-wise classification aspect of the model, then captures the relevancy score of candidate paragraphs using the probability of the "true” token (versus the "false” token). The ensemble stage involves hyperparameter searching to find the optimal weight for each checkpoint. The approach achieved state-of-the-art performance in Task 2 this year, demonstrating the effectiveness of their proposed techniques.
- **IITDLI (3 runs)** [4] has explored sparse retrieval models like BM25, as well as dense retrieval models like zero-shot T5 and a GPT3.5 based reranker.
- **JNLP (3 runs)** [2] used N transformer models, denoted as $M_1, M_2,..., M_N,$ respectively, where each model is associated with a specific loss function. For each query-candidate paragraph pair (q, p), they fed the pair into each of the N models to obtain the corresponding similarity scores $s_1(q,p), s_2(q,p),..., s_N(q,p),$ where each $s_i(q,p)$ represents the similarity score computed by the i-th model. Then they added all $s_i(q,p)$ values from i=1 to N as the final similarity score.
- **NOWJ (3 runs)** [14] relies on BERT and LONGFORMER pre-training models without using any external data. Additionally, they employ an internal data generation method based to overcome the lack of data and enhance the legal case retrieval process.
- **THUIR (3 runs)** [5] implemented the following two lexical matching methods as baselines: BM25 and QLD [17]. BM25 is a classical lexical matching model with robust performance. QLD is another representative traditional retrieval model based on Dirichlet smoothing. Furthermore, contrastive learning loss is employed to fine-tune pre-trained models of different sizes. Finally, they use the above features in an ensemble to produce the final score. Their run with monoT5 [8] placed third, and the run with the ensemble placed fifth.
- **UONLP (1 run)** [3] examined the potential of an agreement-based ensemble model that incorporates two differently pretrained RoBERTa [6] models by assessing their agreement on entailment decisions in order to improve overall performance. The first RoBERTa model was pretrained on a large corpus of Canadian court cases, while the other model was “pre-finetuned” on a corpus of annotated entailment text pairs. Since both models had a different focus in their training data, the goal of the ensemble was to leverage both the strengths of the different models by prioritizing candidate cases that both models would agree upon. Their model was ranked in 9th place in this year’s Task 2 competition.

**Table 2:** Results attained by all teams on the test dataset of task 2

Team	F1	Precision	Recall
CAPTAIN	**0**.**7456**	0.7870	0.7083
CAPTAIN	0.7265	0.7864	0.6750
THUIR	0.7182	0.7900	0.6583
CAPTAIN	0.7054	0.7596	0.6583
THUIR	0.6930	0.7315	0.6583
JNLP	0.6818	0.7500	0.6250
IITDLI	0.6727	0.7400	0.6167
JNLP	0.6545	0.7200	0.6000
UONLP	0.6387	0.6441	0.6333
THUIR	0.6091	0.6700	0.5583
NOWJ	0.6079	0.6449	0.5750
NOWJ	0.6036	0.6569	0.5583
NOWJ	0.5982	0.6442	0.5583
IITDLI	0.5304	0.5545	0.5083
JNLP	0.5182	0.5700	0.4750
IITDLI	0.5091	0.5600	0.4667
LLNTU	0.1818	0.2000	0.1667
LLNTU	0.1000	0.1100	0.0917

### Results and Discussion

The F1 score is used to assess performance in this task. The actual results of the submitted runs by all participants are shown in table 2, from which it can be seen that the CAPTAIN team attained the best results. Among the three submissions from CAPTAIN, two of them were ranked first and second.

THUIR has shown that LEGAL-BERT-base with 110 m parameters has outperformed BERT-large and RoBERTa-large, which indicates that legal-oriented pre-training tasks augment the language model with more legal knowledge and thus achieve better performance. They also showed that more parameters can help language models perform better on legal case entailment tasks. In addition, many participants have tried ensemble methods. UONLP has shown that ensemble methods improved recall while they penalized precision. So, sometimes an ensemble model’s F measure value was worse than that of a single model due to a significant drop in the precision value. In addition, the key to enhancing the performance of the ensemble lies in finding an alternative to the current confidence-based criterion when no agreement can be reached among basic models. JNLP also used an ensemble approach and was able to significantly enhance their performance on this task. In addition, NOWJ tried a weighted ensemble method and showed improved performance. CAPTAIN’s results show that the sequence-to-sequence approach has better performance in textual entailment tasks compared to the BERT re-ranker approach.

Some teams have pointed out the problem of sparse training data, so their ranking method did not achieve satisfactory performance. It may indicate that the answer paragraphs cannot be simply confirmed by information retrieval techniques. Therefore, Task 1 (information retrieval task) and Task 2 (information entailment task) should be approached in a different way. Some experimental results have, perhaps unsurprisingly, shown that more parameters and more legal knowledge contribute to better legal text understanding.

## Task 3—STATUTE LAW INFORMATION RETRIEVAL

### Task Definition

Task 3 requires identification and retrieval of an appropriate subset ($S_1$, $S_2$,..., $S_n$) of Japanese Civil Code Articles from the Civil Code texts for answering a legal bar exam question statement *Q*.

An appropriate subset means that an appropriate entailment system can use that identified subset to judge whether the statement *Q* is true Entails$(S_1, S_2,..., S_n, Q)$ or not Entails$(S_1, S_2,..., S_n, not Q)$.

### Dataset

For Task 3, questions related to the Japanese Civil Code were selected from the Japanese bar exam. We use a part of the Japanese Civil Code that has an official English translation (the number of articles used in the dataset is 768). The training data (the questions and corresponding article pairs) were constructed using previous COLIEE data (996 questions). For the test data, new questions selected from the 2022 bar exam are used (100 questions). 72 questions have a single relevant article and 28 questions have 2 relevant articles.^4^

### Approaches

The following 6 teams submitted their results (16 runs in total). There are two main approaches for the basic IR system component of the task. One is to use a Large Language Model (LLM)-based ranking model. CAPTAIN and JNLP use monoT5 for English. HUKB and CAPTAIN use tohoku BERT^5^ for Japanese. NOWJ uses bert-base-multilingual-uncased^6^ for multilingual settings.

The other approach is the keyword-based approach. HUKB, NOWJ, JNLP, UA use BM25. LLNTU and UA use TF-IDF. Four teams (CAPTAIN, HUKB, JNLP, and NOWJ) combine their approaches using an ensemble strategy to generate final results, using output from IR systems with different settings. Three teams (CAPTAIN, JNLP, and NOWJ) use an ensemble method to obtain results using Japanese and English.

- **CAPTAIN (3 runs)** [7] introduces a simple yet effective method to ensemble model checkpoints in local optima to make a generalized system finally. They assume that each local optimum is biased to some categories, while the target is to build a system that can explore all the categories via sub-models and aggregate the strengths of these submodels. In addition, they uses LLM-based ranking models; Tohoku BERT for Japanese and monoT5 for English. The best performance system uses the ensemble of these two results.
- **HUKB (3 runs)** [16] uses ensembles of keyword-based IR with different settings and LLM-based ranking models using Tohoku BERT.
- **JNLP (3 runs)** [2] uses ensembles of BM25 for Japanese and LLM-based ranking model for English; monoT5.
- **LLNTU (3 runs)** uses an ordinal keyword-based system (TF-IDF) and emphasizes keywords identified by a named entity recognition system.
- **NOWJ (1 run)**^7^ [14] uses a two-stage retrieval system that selects candidates using BM25 and re-ranks the results using an LLM-based ranking model; bert-base-multilingual-uncased for English and Japanese. They use both English and Japanese text to calculate the final score.
- **UA (3 runs)** [11] uses BM25 (UA.BM25), TF-IDF (UA.tfidf) for IR module.

### Results

Table 3 presents the results of evaluating the submitted runs for Task 3 in this study. The official metric employed was the macro-average, which represents the average of scores for each question across all questions, of the F2 score. The F2 score was preferred as it places greater emphasis on recall, given that it constitutes the pre-process for the entailment task. Without the inclusion of relevant articles, the entailment task is meaningless. We use the macro-average to encourage participants to retrieve more candidate articles for difficult queries without compromising the overall F2 score.

4 $$\begin{aligned} F2= & {} \frac{5 \times \text {precision} \times \text {recall}}{4 \times \text {precision} + \text {recall}}. \end{aligned}$$

We also calculate the mean average precision (MAP), and recall at *k* (R_k_: recall is calculated using the top *k* ranked documents as returned documents) using the long ranking list (100 articles).

Table 3 shows the results of the evaluation of the submitted results.

This year, CAPTAIN is the best run among all runs. The top four systems use ensemble settings for the various IR modules, including the LLM-based ranking model. The others use only keyword-based IR. These results confirm the effectiveness of using the LLM-based ranking model.

**Table 3:** Evaluation results of Task 3

TEAM	return	retrieved	F2	Precision	Recall	MAP
CAPTAIN	142	92	**0**.**764**	**0**.**733**	0.800	0.699
CAPTAIN	143	91	0.754	0.723	0.790	0.699
JNLP	194	**98**	0.753	0.652	**0**.**830**	0.717
CAPTAIN	147	91	0.749	0.713	0.785	**0**.**854**
NOWJ	154	90	0.735	0.689	0.775	0.798
HUKB	172	85	0.679	0.634	0.715	0.746
JNLP	174	83	0.669	0.649	0.710	0.693
HUKB	155	81	0.669	0.657	0.690	0.748
JNLP	146	80	0.664	0.672	0.685	0.693
LLNTU	100	74	0.660	0.740	0.650	0.772
HUKB	130	78	0.655	0.685	0.665	0.748
LLNTU	100	71	0.639	0.710	0.630	0.770
UA	109	67	0.570	0.627	0.570	0.661
UA	100	64	0.560	0.640	0.550	0.661
UA	100	64	0.556	0.640	0.545	0.655
LLNTU	100	43	0.386	0.430	0.380	0.515

There are a good number of questions with a single relevant article where all systems can find the relevant article. For 17 questions, all systems can find the relevant article without adding non-relevant articles (precision and recall = 1). For 12 questions, all systems can find the relevant article, but some of the systems add non-relevant articles for the candidates (precision < 1 and recall = 1). In contrast, for answers with multiple relevant articles, there is no question that all systems can find all relevant articles. In addition, there are 4 questions where none of the systems can find the relevant article: 2 of them are questions with 2 relevant articles (R04-13-O, R04-19-E) and 2 of them are questions with 1 relevant article (R04-09-I, R04-12-A).

**Fig. 1:**
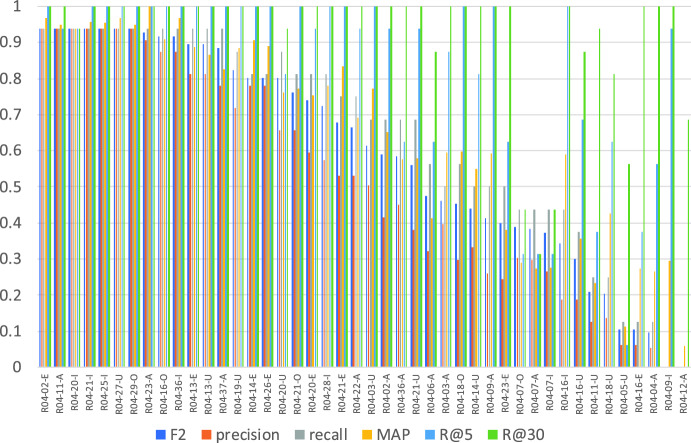
Averages of precision, recall, F2, MAP, R_5, and R_30 for questions with a single relevant article

**Fig. 2:**
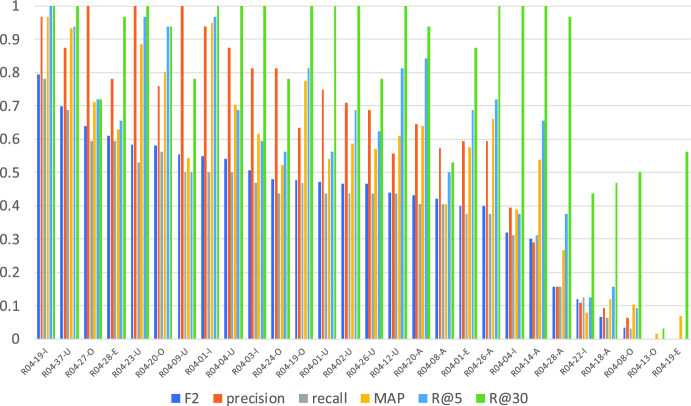
Averages of precision, recall, F2, MAP, R_5, R_10, and R_30 for multiple relevant articles

Figs. 1 and 2 show the average of the evaluation measure of all submission runs for the questions with a single relevant article^8^ and those with multiple relevant articles. As we can see from comparing these two graphs, questions with multiple relevant articles are more difficult than those with a single relevant article. And as indicated by Fig. 2, precision is good compared to recall for the question with multiple relevant articles. This means that most systems succeed in finding the relevant article without adding irrelevant articles, but fail to find the secondary relevant article.

### Discussion

One of the characteristics of difficult questions is the use of anonymized symbols such as "A” and "B” to refer to persons or other entities. There are 40 questions that use such symbols. 3 questions (R04-09-I, R04-12-A, R04-13-O) out of 4 hard questions where no system can find the article using anonymized symbols. Table 4 shows the number of questions for the F2 score (average) that are classified as having an anonymized symbol or not. From this table, we can see that the questions with anonymized symbols are more difficult than those without. The average of F2 score for these questions is 0.500 and for the others it is 0.678.

**Table 4:** Number of questions classified by F2 score and question type

F2	Anonymize	Other
0$-$0.2	5	6
0.2$-$0.4	7	5
0.4$-$0.6	13	9
0.6$-$0.8	2	8
0.8$-$1.0	13	32

Another key factor is the number of relevant articles. R04-09-U is a question where all systems can find one relevant article, but none of the systems can find the secondary relevant article. This question discusses the issue of "Right of way over other land for access by the superficiary of the land.” There are two relevant articles. One is Article 210 which discusses "Right of way over other land for access by the owner of the land” and the other is Article 267 which explains that Article 210 applies, mutatis mutandis, between a superficiary and a landowner. Article 210 shares many keywords with the question and all systems can find the article with precision = 1; however, all systems cannot find Article 267 because most of the keywords in the article are not shared with the question. This is a typical example of secondary relevant articles that are difficult to find by comparing the question and the article one by one. More research is needed to better determine any secondary relevant articles.

Another type of difficult question is the existence of other articles that share common terms. For example, question R04-12-A is about “Statutory Liens for Payment Obligations.” The relevant article is Article 303, which discusses “statutory liens over owner’s claim,” but there is Article 575, which shares keywords, such as "payment” and "obligation,” and many keyword-based IR systems select this article as the relevant one. The LLM-based system can handle the context and select the article related to “statutory liens.” However, it is not easy to identify the relationship between “holder’s own claim” and “payment obligation.” It is also necessary to have a framework to identify such relationships, which is widely used in the discussion of the applicability of the statute.

Another issue is the use of different languages to find the relevant articles. The top three teams (CAPTAIN, JNLP, NOWJ) use both English and Japanese questions to retrieve the relevant articles. The effect of using different languages (English and Japanese) for the COLIEE task was previously discussed in [15]. For example, the Japanese IR system works well when the legal terminology represented by Chinese characters is effective for finding the relevant article. However, there is no guarantee that such Chinese characters are effective. Therefore, the ensemble of results from the multilingual IR system improves the performance of the monolingual IR system in terms of recall. However, it is not common that the user can provide such information in two languages. To encourage the development of stronger methods, it may be better to prohibit the use of questions in both languages. Even in such a case, we can use a machine translation system to have questions in two languages.

## Task 4—Statute Law Textual Entailment and Question Answering

### Task Definition

Task 4 requires the determination of entailment relationships between a given problem sentence and article sentences. Competitor systems should answer “yes” or “no” regarding the given problem sentences and given article sentences. Until COLIEE 2016, the competition focused on a pure entailment task, where t1 (relevant article sentences) and t2 (problem sentence) were given. Due to the limited number of available problems, COLIEE 2017 and 2018 did not retain this style of task. In Task 4 of COLIEE after 2019, we returned to the pure textual entailment task to try and attract more participants and encourage more focused analyses. Participants could use any external data, except that they can not use the test dataset and/or something which could directly contain the correct answers of the test dataset, because this task is intended to be a pure textual entailment task. We also required the participants to make their system reproducible as per an open academic standard, i.e., they should describe which methods and what datasets were used to enable a reproducible result. To encourage deeper analysis, we asked the participants to submit their outputs when using any fragment of the training dataset (H30-R02), in addition to the formal runs. Note that this reproducibility aspect can have a negative impact on the use of black box LLMs like ChatGPT.

### Dataset

Our training dataset and test dataset are the same as for Task 3. Questions related to Japanese civil law were selected from the Japanese bar exam. The organizers provided a data set used for previous campaigns as training data (996 questions) and new questions selected from the 2023 bar exam as test data (101 questions).

### Approaches

We describe approaches for each team as follows, shown as a header format of **Team Name (number of submitted runs)**.

- **AMHR (3 runs)** [1]: **AMHR01** employed 2-shot prompting using the FlanT5-XXL model from Google Research on HuggingFace,^9^ where the shots, balanced by label, were chosen from the training set using a TF-IDF similarity metric for each example at inference time. **AMHR02** used several publicly available models to assemble an ensemble of few-shot prompted models. **AMHR03** employed 6-shot prompting using the GPT-4 model.^10^
- **CAPTAIN (3 runs)** [7]: **CAPTAIN.run1** split each article and query into pairs of (condition, statement) and consider the consensus of the conditions and the statements between the query and an article by Electra.^11^**CAPTAIN.run2** chunked articles to phrases (using an n-gram model), then encoded all phrases and queries using BERT to train a SVM model for classification. This result was combined in an ensemble with **CAPTAIN.run1** to produce the final result. **CAPTAIN.gen** matched pair questions with summaries of relevant articles for classifying the label by BERT.^12^
- **HUKB (3 runs)** [16]: **HUKB1** used Japanese pretrained BERT.^13^**HUKB2** used the Task 3 retrieval system for sub-articles to select appropriate parts of the article, and then applied the same BERT system for generating the final result. **HUKB3** used their BERT-based Task 3 retrieval system for the sub-articles to select the appropriate part of the article and apply the same BERT system for generating their final result. Their systems are almost equivalent to their system submitted for COLIEE 2022.
- **JNLP (3 runs)** [2]: **JNLP** used zero-shot models of LLMs, by gathering all the prompts from the GLUE tasks available in the PromptSource library, selecting 56 prompts. After evaluating the performance of their approach on the provided dataset, they select the prompt that yields the highest accuracy score. During the inference phase, they utilize this prompt to generate the final predicted labels. **JNLP1** used google/flan-t5-xxl model^14^, **JNLP2** used google/flan-ul2 model,^15^**JNLP3** used declare-lab/flan-alpaca-xxl model,^16^ respectively, to run the prompts which were the given problem-article pairs inserted.
- **KIS (3 runs)** [10]: **KIS** extended their previous system which performs data augmentation and ensemble of BERT-based models and rule-based models, in order to integrate LUKE,^17^ the named entity enhanced Transformer. **KIS1** uses the pretrained LUKE model, **KIS2** used a fine-tuned LUKE model for the alphabetical person included dataset, and **KIS3** used another fine-tuned LUKE model without the alphabetical person included dataset.
- **LLNTU (3 runs)**: **LLNTU** used the Disjunctive Union of Longest Common Subsequence, and adjusted them from similarity and length.
- **NOWJ (3 runs)** [14]: **NOWJ** used multi-task model with pre-trained Multilingual BERT^18^ as backbone; **NOWJ.multiv1-en** employed the English data for the training phase, **NOWJ.multi-v1-en** used the Japanese data for the training phase, and **NOWJ.multijp** also used the Japanese data with a different inference strategy.
- **TRLABS (3 runs)**: Their three runs directly use GPT-4 with zero-shot prompting, prompted with IRAC legal reasoning approach (**TRLABS_I**), prompted with TREACC legal reasoning approach (**TRLABS_T**), and no-legal reasoning approach prompted just asked to analyze Hypothesis given the Premise (**TRLABS_D**). Due to the reproducibility issues with GPT-4, these runs are not regarded as formal results.
- **UA (2 runs)**: [11] Their system incorporates the semantic information into the BERT to help the pragmatic reasoning, for natural language inference. **UA_V1** fine-tuned on DeBERTa-small and **UA_V2** fine-tuned on DeBERTa-large model.

### Results and Discussion

Table 5 shows the COLIEE 2023 Task 4 formal run results. The Formal Run (R04) column shows the result of the COLIEE 2023 formal run using the latest Japanese legal bar exam (Year R04). The columns of R02 and R01 are the results using the past formal run datasets, which we required participants to submit, in order to compare different datasets for reference due to the smallness of our datasets. Note that these datasets were already made public as part of our training dataset.

The lower part of the table shows runs with prefixes of “*” to indicate those methods that used external services where its detailed architecture, training datasets, and model weights are not available, resulting in non-reproducible output; such is prohibited in our participation call.

The best runs by team **JNLP** used LLMs in a straightforward way. The second best runs by team **KIS** used BERT and rule-based systems, which is an extension of their previous system, the best of which was in COLIEE 2022. Comparing the results of the past formal run settings (R02 and R01), we found that the rankings switch between these runs from this year’s formal run. In the R01 dataset, the best run was **AMHR01**, which also uses an LLM. These suggest that the required accuracy still depends on the characteristics of each year’s dataset, while LLMs are, at least, comparable or better than the existing models.

A concern with LLMs is that we do not completely grasp what texts are used to train the LLMs; they could include texts very similar or even identical to the COLIEE’s problem/answer texts. This is fine if we simply expect the systems to answer Yes/No in any way, but would not work in general, especially when logical reasoning is required in the statute law, as anticipated in practical use cases.

Another issue to discuss is the reproducibility of the external resources, e.g., OpenAI’s ChatGPT and GPT-4. Some of the teams employed those services, and their performance using GPT-4 showed the same accuracy (0.7822) as that of the winning team **JNLP3**. However, those services could change monthly, weekly, or even daily; and we do not know what dataset was used in their training. The use of such non-reproducible services would not align with our academic intentions.

Still, there remain questions as to what extent those LLM services could solve COLIEE problems. We asked ChatGPT (GPT-4 and GPT$-$3.5 Turbo) to answer the COLIEE problems, using straightforward prompts of “please answer yes or no given the following question:” (in Japanese) with the problem texts as they are, and also asking the main points in its answers. ChatGPT sometimes shows evidence which is inappropriate or wrong even if the Yes/No answer itself is correct. For example, this happens by noting information which is not related to the answer, is almost a direct repetition of the problem text, and which do not address segments noted as “except listed below.” However, GPT-4 worked very well compared to GPT$-$3.5, better than any other LLM solvers, and provided correct main points as far as we manually checked.

It is not clear in what way the GPT-based generative AIs could handle logical reasoning. A possibility is that they can apply superficially similar descriptions which include the use of logical reasoning, so they do not directly handle logic but indirectly reflect the use of logic in existing descriptions and their combinations, i.e., their huge stack of similar contents led to providing approximate answers and marginally related evidence. Because Task 4 is intended to be a pure textual entailment task, superficial similarities without logical reasoning would not make much sense. Thus, we need further investigations about the capability of the generative AIs on logical reasoning. However, as a practical legal application, it can be useful when there are, to some extent, similar contents available as previous existing cases. For our future work, we need new task designs which provide a framework for explainability of results and to evaluate the explainability of the solvers in more practical task settings.

**Table 5:** Evaluation results of submitted runs (Task 4)

Submission ID	L	Formal Run (R04)		R02		R01	
		#	Accuracy	#	Accuracy	#	Accuracy
Total	–	101	–	81	–	111	–
Baseline	–	52	0.5149	43	0.5309	59	0.5315
JNLP3	E	79	0.7822	65	0.8025	72	0.6486
JNLP1	E	76	0.7525	66	0.8148	75	0.6757
JNLP2	E	76	0.7525	63	0.7778	75	0.6757
KIS2	J	70	0.6931	58	0.7160	77	0.6937
KIS1	J	68	0.6733	56	0.6914	74	0.6667
UA_V2	?	67	0.6634	N/A	N/A	N/A	N/A
AMHR01	E	66	0.6535	65	0.8025	79	0.7117
KIS3	J	66	0.6535	54	0.6667	73	0.6577
AMHR03	E	65	0.6436	63	0.7778	49	0.4414
LLNTUdulcsL	J	63	0.6238	42	0.5185	55	0.4955
UA	?	63	0.6238	61	0.7531	67	0.6036
HUKB2	J	60	0.5941	50	0.6173	60	0.5405
CAPTAIN.gen	J	59	0.5842	55	0.6790	65	0.5856
CAPTAIN.run1	E	58	0.5743	41	0.5062	67	0.6036
LLNTUdulcsS	J	57	0.5644	44	0.5432	50	0.4505
HUKB1	J	56	0.5545	41	0.5062	67	0.6036
HUKB3	J	56	0.5545	48	0.5926	61	0.5495
LLNTUdulcsO	J	56	0.5545	44	0.5432	49	0.4414
NOWJ.multi-v1-jp	J	55	0.5446	N/A	N/A	N/A	N/A
CAPTAIN.run2	E	53	0.5248	42	0.5185	67	0.6036
NOWJ.multijp	J	53	0.5248	N/A	N/A	N/A	N/A
NOWJ.multi-v1-en	E	49	0.4851	N/A	N/A	N/A	N/A
AMHR02	E	82	0.8119	66	0.8148	89	0.8018
TRLABS_D	E	79	0.7822	68	0.8395	90	0.8108
TRLABS_I	E	79	0.7822	71	0.8765	87	0.7838
TRLABS_T	E	76	0.7525	71	0.8765	87	0.7838

L: Dataset Language (J: Japanese, E: English), #: number of correct answers. The Baseline answers *No* to all problems

## Conclusion

We have summarized the systems and their performance as submitted to the COLIEE 2023 competition. For Task 1, most teams used traditional IR techniques, LLMs, or a combination of both; the best-performing team was THUIR, which used heuristic pre- and post-processing and learning-to-rank methods. In Task 2, the winning team was CAPTAIN, and they used an approach based on the pre-trained monoT5 sequence-to-sequence model, which is fine-tuned with hard negative mining and ensembling techniques to achieve an F1 score of 0.7456. For Task 3, the best F2 score is 0.757 by CAPTAIN that uses monoT5 for English and Tohoku BERT for Japanese and ensemble their results. Lastly, for Task 4, the best accuracy score was 0.7822 by JNLP3 using LLMs. We intend to further continue to improve dataset quality in future editions of COLIEE so the tasks more accurately represent real-world problems.

After ten years of COLIEE, it seems that a reasonable direction forward is to design an evolution of the competition that provides more emphasis on explicit error analysis (what didn’t work), and on the integration of explainability in all systems, to understand errors and how they can be addressed in general (e.g., how to “debug” LLMs).

## Footnotes

1 "Notice” is a legal technical term that denotes a legal case description that is considered to be relevant to a query case.

2 For a description of the previous Task 1 formulation, please see the COLIEE 2020 summary [12].

3 https://huggingface.co/castorini/monot5-large-msmarco and https://huggingface.co/castorini/monot5-large-msmarco-10k.

4 We found an English translation problem for the question of R04-04-E and exclude the question from official evaluation.

5 https://github.com/cl-tohoku/bert-japanese-whole-word-ma.

6 https://huggingface.co/bert-base-multilingual-uncased.

7 Due to a system error, two runs were withdrawn from the official evaluation.

8 Due to space limitations, we exclude 29 questions, all systems can find all relevant articles (recall = 1).

9 https://huggingface.co/google/flan-t5-xxl.

10 https://openai.com/blog/openai-api.

11 https://huggingface.co/google/electra-base-discriminator.

12 https://huggingface.co/cl-tohoku/bert-base-japanese-whole-word-masking.

13 https://huggingface.co/cl-tohoku/bert-base-japanese-whole-word-masking.

14 https://huggingface.co/google/flan-t5-xxl.

15 https://huggingface.co/google/flan-ul2.

16 https://huggingface.co/declare-lab/flan-alpaca-xxl.

17 https://huggingface.co/studio-ousia/luke-japanese-base-lite.

18 https://huggingface.co/bert-base-multilingualuncased.
